# Does chemoradiotherapy benefit elderly patients with esophageal squamous cell cancer? A propensity-score matched analysis on multicenter data (3JECROG R-03A)

**DOI:** 10.1186/s12885-019-6461-z

**Published:** 2020-01-15

**Authors:** Mingqiu Chen, Xiaohong Liu, Chun Han, Xin Wang, Yidian Zhao, Qingsong Pang, Xinchen Sun, Gaofeng Li, Kaixian Zhang, Ling Li, Xueying Qiao, Yu Lin, Junqiang Chen, Zefen Xiao

**Affiliations:** 1 Department of Radiation Oncology, Fujian Cancer Hospital & Fujian Medical University Cancer Hospital, No. 420, Fumalu Road, Jinan District, Fuzhou City, Fujian Province People’s Republic of China; 2 The Graduate School, Fujian Medical University, Fuzhou, 350122 Fujian China; 3 Department of Radiation Oncology, the Fourth Hospital of Hebei Medical University, Shijiazhuang, 050011 China; 4 Department of Radiation Oncology, National Cancer Center/National Clinical Research Center for Cancer/Cancer Hospital, Chinese Academy of Medical Sciences and Peking Union Medical College, Beijing, 100021 China; 5 Department 4th of Radiation Oncology, Anyang Cancer Hospital, Anyang, 455000 China; 6 Department of Radiation Oncology, Tianjin Medical University Cancer Institute and Hospital/National Clinical Research Center for Cancer, Tianjin, 300060 China; 7 Department of Radiation Oncology, the First Affiliated Hospital of Nanjing Medical University, Nanjing, 210029 China; 8 Department of Radiation Oncology, Beijing Hospital, National Center of Gerontology, Beijing, 100730 China; 9 Department of Oncology, Tengzhou Central People’s Hospital, Tengzhou, 277599 China

**Keywords:** Concurrent chemoradiotherapy, Elderly, Esophageal squamous cell carcinoma, Survival

## Abstract

**Background:**

The aim of the present study was to assess the efficacy of concurrent chemoradiotherapy (CRT) or radiotherapy alone (RT-alone) in elderly patients with esophageal squamous cell carcinoma (ESCC).

**Methods:**

The clinical data of patients with ESCC treated with RT-alone or CRT were collected and retrospectively reviewed. The 1-, 3- and 5-year overall survival (OS) rates and the clinical characteristics correlated with survival were analyzed statistically. Propensity score matching (PSM) analyses were used to compensate for differences in baseline characteristics between the CRT and RT-alone groups to confirm the survival difference.

**Results:**

A total of 729 patients fulfilling the inclusion criteria were reviewed. Diabetes, primary tumor volume (pTV), primary tumor location (pTLo), clinical T stage,(cT) clinical N stage (cN), clinical M stage (cM) and short-term response to RT were independent factors influencing OS (*P* = 0.002–0.044). The 5-year OS rate was 26.6, 26.0 and 30.1% in the whole cohort, RT-alone and CRT groups, respectively. The survival difference between RT alone and CRT was not significant before or following PSM. Compared with the corresponding subgroups treated with RT alone, CRT significantly benefited patients with diabetes (*P* = 0.003), cT4 (*P* = 0.030) and cN0 (*P* = 0.049), whereas no benefit was identified between CRT and RT alone in the other subgroups, including cT1–3, cN1, cM, pTLo, pTV, age and gender.

**Conclusions:**

CRT with the current chemotherapy regimens may not improve the survival of elderly ESCC patients compared to RT-alone, except in patients with cT4 stage, cN0 stage or diabetes. However, due to the limitation of the retrospective nature of the current study, further clinical trials are required for confirmation.

## Background

Esophageal cancer (EC) is the seventh most common cancer, with an estimated 572,000 new cases and 509,000 deaths in 2018 [1]. EC is rare among young individuals and increases in incidence with age, peaking in the seventh and eighth decades of life [2]. With the increased age of the general population, the number of elderly patients with EC is expected to increase in the foreseeable future.

Esophagectomy is considered an effective treatment for EC. However, due to physiological limitations and high operative morbidity and mortality, elderly patients with EC are reluctant to undergo surgical procedures [3]. For patients with unresectable or medically inoperable EC, concurrent chemoradiotherapy (CRT) is considered an optimal alternative. Due to intolerance to the acute toxicities of standard CRT [4], most elderly patients require an altered treatment strategy. The efficacy of CRT in elderly patients with EC had not been established, due to conflicting and inconclusive results from previous studies [5–7].

In the current study, the clinical data of elderly patients with esophageal squamous cell cancer (ESCC) initially treated with radiotherapy alone (RT-alone) or CRT were collected retrospectively to explore the role of CRT in elderly patients with ESCC.

## Methods

### Patient selection criteria

This retrospective study was approved by Fujian Cancer Hospital & Fujian Medical University Cancer Hospital (No.SQ2019–037-01) and National Cancer Center/National Clinical Research Center for Cancer/Cancer Hospital (NCC2016–06) Institutional Review Board. All patients provided written informed consent prior to treatment, and all information was anonymized prior to analysis.

The eligibility and exclusion criteria for the present retrospective study were similar to those of our previous study [8], except patient age. In brief: histologically proven ESCC; > 70 years old and life expectancy ≥3 months; pretreatment assessment available to define the clinical stage and to assess the suitability for RT-alone or CRT; Eastern Cooperative Oncology Group scoring (ECOG) ≤3; clinical stage of T_any_N_any_M_0_ or M_1_ only with supraclavicular lymph node metastasis (SLNM); no neo- or adjuvant chemotherapy; no post-RT salvage surgery performed; and sufficient follow-up data available for survival assessment.

The clinical TNM stage was determined according to the 7th American Joint Committee on Cancer (AJCC) TNM staging system, based on computed tomography (CT) scanning data of T, N and M stage.

### Radiotherapy and concurrent chemoradiotherapy

All enrolled patients were administered radical CRT or RT-alone. The RT technology consisted of three-dimensional conformal radiation therapy (3D-CRT) or intensity-modulated radiation therapy (IMRT). The concurrent chemotherapy (CC) time intervals and dose intensities were reported in our previous study [9].

The targets, including gross tumor volume, clinical target volume and organs at risk of radiotherapy, the target dose and the dose limitations of organs at risk were defined and adjusted as described in our previous study [9].

### Surveillance and statistical analysis

The follow-up schedule for patients was as previously reported [9]. In brief, patients were assessed every 3 months for the first 2 years after RT, every 6 months for the next 3 years, and then once annually. All patient outcomes were evaluated in March 2018. The primary endpoint of the current study was overall survival (OS), which was calculated from the date of RT completion to the date of mortality or final follow-up.

Data were analyzed using SPSS, version 24.0 (IBM Corp., Armonk, NY, USA). Survival curves were generated using the Kaplan-Meier estimator method and compared using the log-rank test. Univariate and multivariate analyses, including age, gender, frequently preexisting chronic comorbidities, treatment modality, primary tumor length (pTL), primary tumor location (pTLo), primary tumor volume (pTV), cT stage, cN stage, cM stage, cTNM stage, radiation dose of GTV, radiotherapy technology, cycles and regimens of CC, were performed using the Cox proportional hazards model. Confidence intervals (CI) represented 95% lower and upper bounds. *P* ≤ 0.05 was considered to indicate a statistically significant difference.

According medical history records, the following four diseases were considered the most frequently preexisting chronic comorbidities: hypertension, cardiovascular disease, pulmonary disease and diabetes.

Propensity score matching (PSM) analyses were used to compensate for differences in baseline characteristics between the CRT and RT-alone groups to confirm the survival difference. First, the Cox hazard model was utilized to determine all available patients and variables correlated with OS by univariable analyses. Next, all the unbalanced variables that were statistically significantly correlated with OS were adjusted by PSM with a match tolerance value at 0.1 [10]. Pearson’s χ^2^ test or an independent samples t-test was subsequently performed to compare the differences between the CRT and RT-alone groups after matching.

## Results

### Patient characteristics

Between September 1, 2004 and December 31, 2015, a total of 961 patients with ESCC treated with RT were reviewed. A total of 729 patients fulfilled the inclusion criteria, of whom 133 (18.2%) patients were administered with CRT and 596 (81.8%) patients were treated with RT-alone. The clinical characteristics of patients are summarized in Table 1.

**Table 1:** Clinical characteristics of pre- and post-matched patients

Characteristics	Pre-PSM				Post-PSM			
	CRT	RT-alone	*χ2*	*P*	CRT	RT-alone	*χ2*	*P*
Gender (n)			0.141	0.708			4.607	0.042
Male	84	366			76	91		
Female	49	230			41	26		
Mean age (year)	73.3 ± 3.58	76.2 ± 4.39		< 0.001	73.8 ± 3.7	73.9 ± 3.8		0.875
ECOG (n)			29.776	< 0.001			6.838	0.078
0	15	208			15	23		
1	46	156			42	38		
2	66	203			57	46		
3	6	29			3	10		
Preexisting chronic comorbidities (n)								
Hypertension								
yes	33	207	4.845	0.032	32	28	0.359	0.654
no	100	389			85	89		
Cardiovascular disease								
yes	16	78	0.108	0.886	14	22	2.101	0.204
no	117	518			103	95		
Pulmonary disease								
yes	14	57	0.115	0.747	13	20	1.729	0.26
no	119	539			104	97		
Diabetes								
yes	12	69	0.718	0.449	11	13	0.186	0.83
no	121	527			106	104		
Tumor location (n)			10.204	0.037			4.728	0.316
Cervical	10	19			9	4		
Upper	40	137			30	39		
Middle	51	288			46	47		
Lower	32	150			32	26		
EGJ	0	2			0	1		
Mean primary tumor length (cm)	5.50 ± 2.30	5.38 ± 2.27		0.564	5.5 ± 2.1	5.7 ± 2.4		0.426
Mean primary tumor volume (cm3)	50.82 ± 34.6	44.91 ± 33.14		0.255	50.2 ± 35.4	52.8 ± 32.4		0.56
Clinical T stage (n)			8.959	0.03			3.411	0.333
T1	0	10			0	3		
T2	14	113			13	10		
T3	56	245			53	52		
T4	63	228			51	52		
Clinical N stage (n)			0.14	0.769			0.018	1
N0	51	239			46	45		
N1	82	357			71	72		
Clinical M stage (n)			13.287	0.001			0.186	0.83
M0	114	564			106	104		
M1	19	32			11	13		
Primary T GTV Dose (cGy)	6120 ± 253	6067 ± 334		0.084	6105 ± 249	6052 ± 389		0.212

Cardiovascular disease: coronary artery disease [CAD] and atrial fibrillation [AF]

Pulmonary disease: chronic obstructive pulmonary disease [COPD] and asthma

*EGJ* Esophagogastric junction

*PSM* Propensity score matching

A median number of 2 (range, 1–4) cycles of CC were administered to CRT patients. The regimens of CC included: single-agent fluoropyrimidine (5-fluorouracil, tegafur, carmofur or capecitabine, *n* = 16) or cisplatin (*n* = 24) or taxane (*n* = 12); dual-agent platinum compound (cisplatin, lobaplatin, nidaplatinum or oxaliplatin) plus fluoropyrimidine (5-fluorouracil or capecitabine; PF) (*n* = 42) or a platinum compound plus taxane (paclitaxel or docetaxel; TP) (*n* = 4); and dual- to single-agent (initially with dual-agent and then with single-agent, *n* = 35).

### Treatment failure and survival analysis in the entire cohort

At the last follow-up in March, 2018, 201 patients remained alive and 528 patients had died, of whom 266 (50.3%) patients had succumbed to primary or locoregional tumor relapse, 132 (25.0%) patients to distant metastasis, and 24 (4.6%) to both locoregional and distant metastasis; 20 (3.8%) had died from treatment complications (18 patients in RT-alone and two patients in CRT, respectively), 50 (9.5%) patients had died of non-tumor disease, and 36 (6.8%) patients had succumbed to unknown causes.

**Fig. 1:**
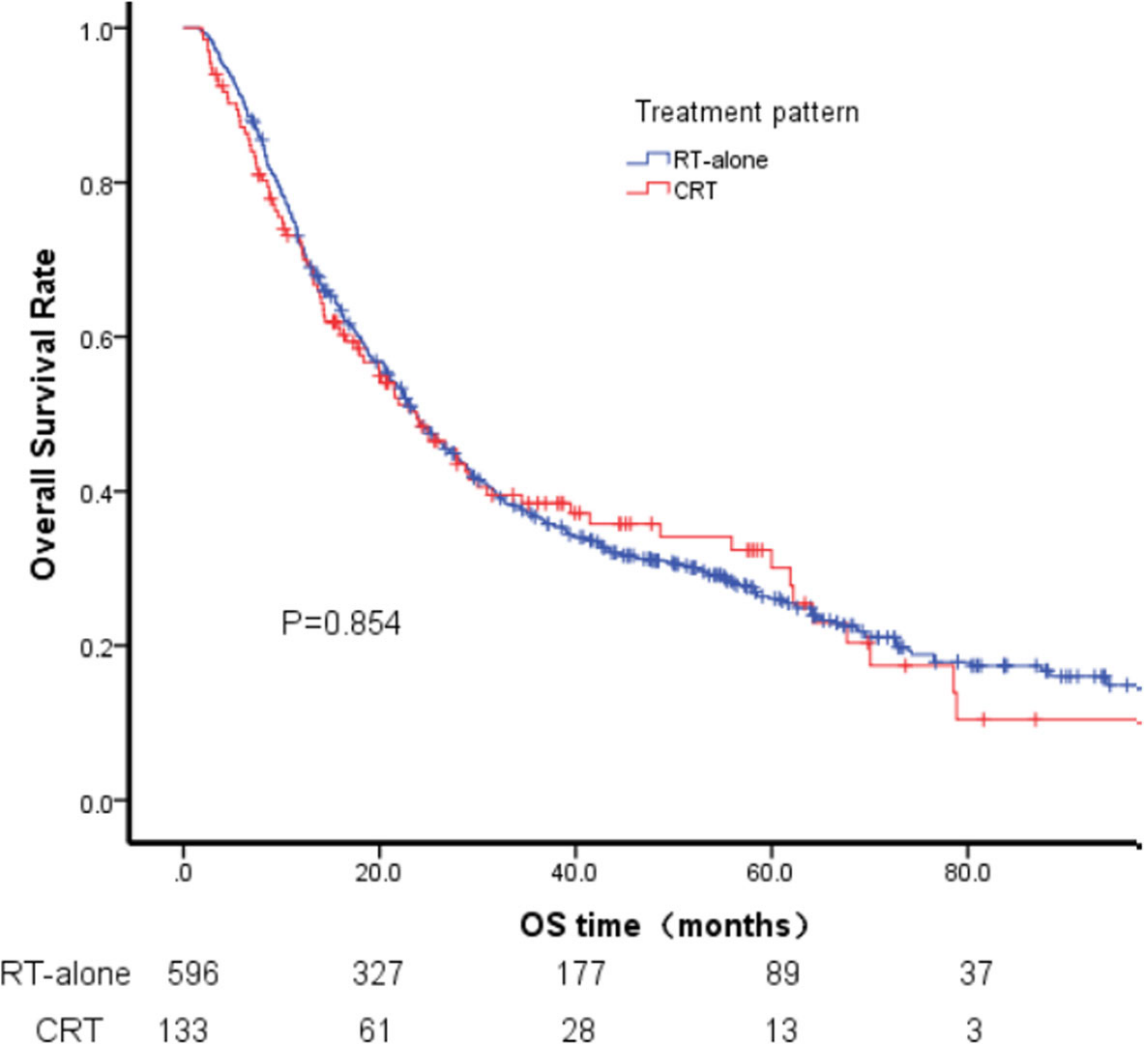
Survival between CRT and RT-alone across the whole cohort

The median follow-up time in the entire cohort and in the surviving patients was 21.7 (1.6–141.2) and 50.8 (3.3–141.2) months, respectively. The 1-, 3- and 5-year OS rate in the entire cohort, CRT and RT-alone groups are presented in Table 2. The survival difference between CRT and RT-alone was not significant (*P* = 0.854; Fig. 1).

**Table 2:** Overall survival rate in the various subgroups and failure pattern in the entire cohort

Overall survival rate (%)	1- year	3- year	5- year	*P*
Treatment				0.854
RT-alone	71.9	36.6	26	
CRT	72.4	38.5	30.1	
Whole	72	36.9	26.6	
Diabetes				0.035
yes	66.5	24.1	16	
no	72.6	38.4	27.8	
pTLo				
upper	77.2	46.9	33.5	0.001
middle	69.5	32.6	21.5	0.006
lower	69.5	33.8	28.2	0.829
pTV (cm^3^)				< 0.001
≤ 32	79.8	46.7	36.1	
> 32	66.8	31	21.2	
cT				
1	90	80	70	0.21
2	75.6	40	33.7	0.16
3	75.9	36.7	28.5	0.1
4	64.9	33.8	20.4	
cN				0.001
0	77.2	46.8	32.7	
1	68.1	30.4	22.5	
cM				< 0.001
0	72.8	38.2	27.4	
1	56.6	16.9	16.9	
Failure Pattern (n)	Total	RT	CRT	*P*
locoregional	266	226	40	0.209
distant	132	103	29	
locoregional and distant	24	20	4	
treatment complication	20	18	2	
non-tumor disease	50	41	9	
unknown causes	36	34	2	

*pTLo* Primary tumor location

*pTV* Primary tumor volume

Univariate and multivariate analyses in the entire cohort indicated that diabetes (*P* = 0.044), pTV (*P* = 0.026), pTLo (*P* = 0.004), cT stage (*P* = 0.028), cN stage (*P* = 0.023), cM stage (*P* = 0.002) and short-term response to RT (*P* = 0.003) were independent factors influencing OS (Table 3).

**Table 3:** Prognostic factors by univariate and multivariate analyses

	Pre-PSM				Post-PSM	
	univariate analyses		multivariate analyses		multivariate analyses	
	*P*	HR(95% CI)	*P*	HR(95% CI)	*P*	HR(95% CI)
Gender	0.184	0.886 (0.742–1.059)				
Age	0.418	1.008 (0.989–1.028)				
ECOG	0.796	1.013 (0.921–1.113)				
Hypertension	0.999	1.000 (0.834–1.199)				
Cardiovascular disease	0.806	1.032 (0.805–1.323)				
Pulmonary disease	0.795	1.038 (0.782–1.379)				
Diabetes	0.036	1.326 (1.019–1.726)	0.044	1.314 (1.007–1.714)	0.028	1.643 (1.056–2.555)
Tumor location	0.006	1.160 (1.044–1.289)	0.004	1.174 (1.052–1.311)		
Primary tumor length	0.001	1.063 (1.024–1.102)				
Primary tumor volume	< 0.001	1.063 (1.024–1.102)	0.026	1.003 (1.000–1.006)		
Clinical T stage	0.001	1.207 (1.079–1.351)	0.028	1.146 (1.015–1.295)	0.028	1.270 (1.026–1.571)
Clinical N stage	0.001	1.351 (1.131–1.613)	0.023	1.237 (1.030–1.485)	0.001	1.673 (1.223–2.287)
Clinical M stage	< 0.001	1.825 (1.308–2.545)	0.002	1.703 (1.209–2.399)	0.001	1.965 (1.295–2.983)
GTV dose	1.398	1.000 (1.000–1.000)				
Technique	0.676	0.964 (0.811–1.146)				
Treatment pattern	0.854	1.022 (0.811–1.288)				
Short-term response to RT	0.001	1.263 (1.095–1.457)	0.003	1.243 (1.076–1.435)		

*PSM* Propensity score matching

*ECOG* Eastern Cooperative Oncology Group scoring

### Survival benefit of CRT in various subgroups following PSM

To balance bias between CRT and RT-alone due to the retrospective nature of this study, PSM, based on the clinical baseline characteristics including gender, age, ECOG, pTL, pTV, pTLo, cT, cN, cM and four chronic comorbidities, was conducted. Following PSM, a total of 234 events were identified in both the matched CRT and RT-alone groups, with 117 patients in each group.

Following PSM, no significant differences in clinical characteristics were identified between the two matched groups, with the exception of gender (Table 1), which did not influence patient survival in the subsequent univariate and multivariate analyses. Univariate and multivariate analyses in the PSM patients demonstrated that only diabetes (*P* = 0.028), cT (*P* = 0.028), cN (*P* = 0.001) and cM (*P* = 0.001) were independent prognostic factors, while pTV, pTLo and short-term response to the RT did not independently affect patient survival.

**Fig. 2:**
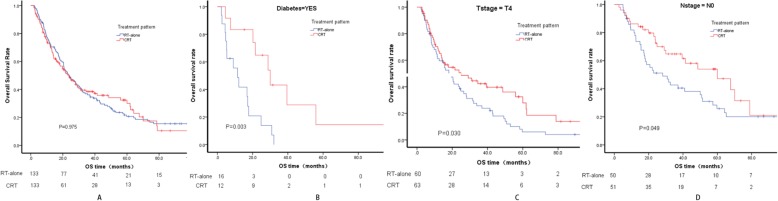
Survival between CRT and RT-alone following PSM (**a**); Survival between CRT and RT-alone in diabetes (**b**), in cT4(**c**), and in cN0 (**d**) following PSM

Following PSM, the OS differences were still not significantly different between CRT and RT-alone (Fig. 2a). To identify patients who may benefit from CRT, exploratory analyses were conducted among various patient subgroups following PSM. The results indicated that compared with the corresponding subgroups treated with RT-alone, CRT significantly benefited patients with diabetes (*P* = 0.003), cT4 (*P* = 0.030) and cN0 (*P* = 0.049) (Fig. 2b, c, d). By contrast, no benefit was identified between CRT and RT-alone in the other subgroups, including cT1–3, cN1, cM stage, pTLo, pTV, age and gender.

## Discussion

Due to physiological limitations and tolerance to the toxicity of aggressive treatment, elderly patients with ESCC are usually given palliative care to prevent deterioration of the general condition of patients. However, cumulative studies have proven that elderly patients with EC achieve long-term survival from curative treatment, such as radical esophagectomy or definitive RT or CRT [11–13]. Alberto et al reported that short- and long-term outcomes after esophagectomy for EC in patients older than 70 years are comparable with those of their younger counterparts [14]. Xu et al reported that, compared to young patients with similar prognostic status, the elderly population exhibits similar long-term survival following definitive CRT [15]. In the current study, which enrolled patients aged over 70 who were treated with RT or CRT, the 5-year OS rate of the whole cohort was similar to that of our previous studies, which enrolled patients aged under 70 (with an OS rate of 27.2% at 5 years) [8]. The results confirmed that old age should not be a contraindication for curative treatment in patients with EC.

Although advanced age is not a contraindication for aggressive treatment, data on whether CRT is superior to RT alone for patients with unresectable or medically inoperable ESCC are scarce, and the efficacy of definitive CRT has not been established [16]. Zhao et al reported that CRT with platinum and 5-FU is well-tolerated and more effective than RT alone for elderly patients older than 75 years with locally advanced ESCC [17]. In the current study, the survival rates between the CRT and RT-alone groups were not significantly different, whether in pre- or post-PSM. The impact of treatment complications on survival is often a common explanation for this discrepancy. However, the treatment-related mortality rate between RT-alone and CRT was not found to be significantly different in the current study. Therefore, for elderly patients with ESCC, combined treatment of RT with a contemporary regimen of chemotherapy should be conducted cautiously [18].

Other potential reasons for the inconsistency are the inadequate intensity of chemotherapy and unified chemotherapy regimens in the current study. However, to date, the appropriate doses and schedules of chemotherapy for these patients are yet to be determined [19]. Several studies had proven that dual-agent therapy achieved superior survival than single-agent CRT for elderly patients with ESCC [20, 21]. Zhao et al demonstrated that CRT with a single agent in elderly patients had a similar survival benefit compared to CRT with a dual agent, accompanied by less toxicity [22]. Similar to Zhao et al, in the current study, the survival of patients treated with CRT with various CC regimens or CC cycles did not alter significantly. The aforementioned results indicate that the efficacy of CC in patients treated with RT is debatable, and novel drug treatment options with lower toxicity and higher efficacy must be developed for elderly patients with ESCC [23, 24].

Despite the controversies, some studies have argued that CRT should be considered for a certain subgroup rather than for all elderly ESCC patients. Zhang et al reported a single-center retrospective study demonstrating that, compared with RT-alone, only elderly ESCC patients aged 65–72, and not patients older than 72 years, benefited from CRT in terms of survival [5]. To identify which subgroup of patients may benefit from CRT, exploratory subgroup analyses basing on the potential factors influencing prognosis were performed in the present study. The results demonstrated that, compared with RT alone, CRT did not confer a survival benefit to elderly patients in the various subgroups, except cT4, cN0 or diabetic patients.

The T category of the current AJCC staging system is based on anatomical information related to primary tumor invasion of the esophageal wall. Although CRT was considered an optimal treatment for unresectable EC, its feasibility and effectiveness for T4 tumors, which are defined by invasion of the adjacent structures and are not indicated for surgery, is still unclear, and a high incidence of esophageal perforation has been reported after standard CRT for T4 tumors [25]. Previous studies have reported the use of a low-dose concurrent chemotherapy regimen to obtain the maximal radiosensitizing effect, which may improve patient survival without the rapid depopulation of massive T4 tumors and perforation caused by full-dose chemotherapy [26–28]. The current study, similar to Nishimura et al [28], demonstrated that CRT compared to RT alone achieved superior survival in cT4 patients. The results suggested that, even in elderly patients, patients with more advanced disease should be administered more aggressive treatments to improve their survival.

Lymph node metastasis (LNM) is a poor prognosis factor in patients with ESCC and an indicator that more intensive treatment may be required [29, 30]. Numerous studies in non-elderly patients have found that for patients with LNM, CRT can achieve a greater survival benefit than RT-alone [31]. However, the efficacy of CC in elderly ESCC patients with LNM treated with RT had not been confirmed. The current study took the lead in discussing this topic. Unfortunately, contrary to the non-elderly patients, the current study found that whether in the entire cohort or in the PSM patients, compared with RT-alone, CRT benefited survival only in N0 patients, and not in N1 patients who were hypothesized to benefit from CC. The inaccurate division of the cN stage into N0 and N1 may have explained the results of the current study. The accurate determination of cN stage based on a CT scan image is crucial for the re-evaluation of the role of CRT in elderly patients with ESCC with LNM.

SLNM is still considered a distant metastatic disease even in the newest 8th edition of the AJCC TNM staging system [32], and palliative treatment is recommended by the NCCN guidelines. Recently, several studies have argued that SLNM does not constitute an important independent prognostic factor for patients treated with CRT [33, 34]. Our previous data demonstrate that the OS of patients with SLNM(+) is superior to those with cN3 SLNM(−), and is similar to those with cN1 SLNM(−) or cN2 SLNM(−), but inferior to cN0 SLNM(−), confirming that SLNM should be treated with curative intent as a regional, rather than a distant, disease in patients with ESCC when treated with CRT [35]. However, in the current study, CRT failed to demonstrate a survival advantage in patients with SLNM compared to RT-alone whether in pre- or post-PSM patients. The enrolled sample of SLNM patients was too small in the current study, which may have been the reason for the lack of statistical differences.

Chronic comorbidities are thought to affect cancer patient outcomes [36–38] and impact treatment decisions. He et al reported that certain comorbidities (hypothyroidism/levothyroxine) affect EC-specific survival in EC patients treated with CRT [39]. Barone et al reported that patients diagnosed with cancer who have preexisting diabetes are at increased risk for long-term, all-cause mortality compared with those without diabetes [37]. It is generally recognized that elderly patients are more frequently affected by different chronic comorbidities. However, the influence of chronic comorbidities on the survival of patients with ESCC treated with RT or CRT had not been reported. Similar to Barone et al [37], in the current study, among the four common chronic comorbidities, diabetes was the only one identified to be an negative factor affecting patient prognosis, whether in the entire cohort or the PSM patients. Further subgroup analysis found that compared to RT-alone, CRT improved patient survival significantly in diabetic patients, but not in non-diabetic patients. Certainly, despite the survival benefits, the greater risk posed by more aggressive treatment in elderly patients with chronic comorbidities must be kept in mind in clinical practice [38].

## Conclusions

Our results demonstrated that CRT with the current chemotherapy regimens may not improve the survival of elderly ESCC compared to RT-alone, except in patients with cT4 or cN0 or diabetes. However, the retrospective nature of the current study, for example the limited records of treatment-related toxicities, the imbalances between the patient characteristics, the diverse chemotherapy regimens, the suboptimal assessment of staging by CT scanning and the cancer-specific comprehensive geriatric assessment tool [40] make it difficult for us to extend our investigation to confirm the results. Thus, further clinical trials are required to evaluate the efficacy of CRT in elderly ESCC patients.

## Supplementary information

**Additional file 1:** Data of 729 elderly patients with esophageal squamous cell cancer.

## Data Availability

Data were applicable to this article in Additional file 1.

## Abbreviations

3D-CRT: Three-dimensional conformal radiation therapy
AJCC: American Joint Committee on Cancer
CC: Concurrent chemotherapy
CI: Confidence intervals
cM: Clinical M stage
cN: Clinical N stage
CRT: Concurrent chemoradiotherapy
cT: Clinical T stage
CT: Computed tomography
EC: Esophageal cancer
ECOG: Eastern Cooperative Oncology Group scoring
ESCC: Esophageal squamous cell carcinoma
GTV: Gross tumor volume
IMRT: Intensity-modulated radiation therapy
LNM: Lymph node metastasis
OS: Overall survival
PSM: Propensity score matching
pTL: Primary tumor length
pTLo: Primary tumor location
pTV: Primary tumor volume
RT-alone: Radiotherapy alone
SLNM: Supraclavicular lymph node metastasis
